# Renal consequences of complex abdominal wall reconstruction

**DOI:** 10.1007/s10029-026-03723-6

**Published:** 2026-05-22

**Authors:** Lucas Maciel de Almeida Corrêa, Luiggi Kevin Virgino Brandão, Gabriel Rian Mazur, Clara Belo Gamon Santiago, Alexandre de Assis Barbosa, Ivan Felipe Dutra Júnior, Giovana Custódio Molinari, Camilla Cristina Silva Fernandes

**Affiliations:** 1 Faculdade de Medicina de São José do Rio Preto (FAMERP), Hospital de Base de São José do Rio Preto, Avenida Brigadeiro Faria Lima, 5416, Vila São Pedro, São José do Rio Preto, SP 15090-000 Brazil; 2 Centro Universitário Uninorte (UNINORTE), BR 364, Km 02, Alameda Alemanha, 200, Jardim Europa, Rio Branco, Acre 69915-901 Brazil; 3 Pontifícia Universidade Católica do Paraná (PUCPR), Rua Imaculada Conceição, 1155, Prado Velho, Curitiba, Paraná 80215-901 Brazil; 4 Universidade do Estado de Mato Grosso do Sul (UEMS), Avenida Dom Antônio Barbosa, 4155, Bairro Santo Amaro, Campo Grande, Mato Grosso do Sul 79115-898 Brazil; 5 Universidade do Estado da Bahia (UNEB), Rua Silveira Martins, 2555, Cabula, Salvador, Bahia 41150-000 Brazil

**Keywords:** Abdominal wall reconstruction, Ventral hernia, Acute kidney injury, Chronic kidney disease, Intra-abdominal hypertension, Loss of domain

## Abstract

**Purpose:**

To review the renal consequences of complex abdominal wall reconstruction (AWR) and examine how reconstructive mechanics, intra-abdominal pressure, perioperative fluid strategy, and baseline renal reserve influence postoperative kidney outcomes.

**Methods:**

This focused narrative review searched PubMed/MEDLINE and Embase for studies published through March 2026 using terms related to abdominal wall reconstruction, complex ventral hernia repair, loss of domain, component separation, transversus abdominis release, intra-abdominal pressure, abdominal compartment syndrome, acute kidney injury, chronic kidney disease, renal dysfunction, and perioperative renal outcomes. Reference lists of key studies were also screened manually.

**Results:**

Postoperative acute kidney injury after complex AWR is not uncommon and appears to cluster in patients with greater reconstructive intensity, particularly those undergoing large ventral hernia repair, major visceral reintegration, or transversus abdominis release. The available literature supports a clinically useful framework in which renal vulnerability after AWR reflects the interaction of pressure-related stress, hemodynamic and fluid-related factors, and limited baseline renal reserve. In selected patients, postoperative kidney injury may extend beyond the index admission.

**Conclusion:**

Complex AWR should be understood not only as an anatomic reconstruction but also as a physiologically demanding operation in which renal dysfunction may signal meaningful perioperative stress. For abdominal wall surgeons, this perspective supports more deliberate interpretation of postoperative oliguria, greater awareness of pressure-mediated organ dysfunction, and closer renal follow-up in high-risk patients.

## Introduction

Complex abdominal wall reconstruction (AWR) is usually framed by surgeons in terms of defect size, loss of domain, component separation, transversus abdominis release (TAR), respiratory consequences, wound morbidity, and recurrence [1–3]. Within this surgical framework, however, postoperative renal dysfunction has received comparatively little attention, despite being one of the clearest physiologic signals that reconstruction has imposed a meaningful systemic burden [1–3]. The problem is therefore not whether kidney risk exists, but that it has not been consistently incorporated into how complex AWR morbidity is interpreted by the abdominal wall surgeon [2, 4, 5]. In this setting, renal dysfunction should be viewed less as an incidental laboratory abnormality and more as a clinically relevant marker of pressure-related and hemodynamic stress after reconstruction [4–6].

This under-recognition is now difficult to sustain [4, 5]. Shelby et al. reported acute kidney injury (AKI) in 21.2% of patients undergoing complex AWR and identified perioperative fluid management as a potentially modifiable domain, although not with intervention-level proof of causality [4]. Messer et al. extended the discussion beyond the index admission, documenting postoperative AKI in 14.2%, new-onset chronic kidney disease (CKD) in 6.9%, and CKD progression in 19.6%, while also showing postoperative AKI to be independently associated with later renal deterioration [5]. Taken together, these data suggest that renal dysfunction after complex AWR should not be dismissed as a transient biochemical event; rather, it may identify the subgroup in whom reconstructive intensity, altered abdominal mechanics, and limited host reserve converge to produce both early and longer-term risk [4, 5, 7].

Accordingly, complex AWR should be regarded not only as a technically demanding abdominal wall operation, but also as a perioperative physiologic stress test in which reconstructive mechanics, abdominal compliance, fluid strategy, and baseline renal reserve interact to shape postoperative outcomes [2, 4, 5, 8]. This review integrates the emerging clinical evidence with a procedure-specific physiological framework and discusses the implications for risk stratification, perioperative interpretation, and future study design in complex ventral hernia repair [2, 4, 5].

## Approach to the literature

Because renal outcomes after complex abdominal wall reconstruction are addressed in a limited and heterogeneous literature, this article was designed as a focused narrative review rather than a formal systematic review [2, 4, 5]. PubMed/MEDLINE and Embase were searched for studies published up to March 2026 using combinations of controlled vocabulary and free-text terms related to abdominal wall reconstruction, complex ventral hernia repair, loss of domain, component separation, transversus abdominis release, intra-abdominal pressure, intra-abdominal hypertension, abdominal compartment syndrome, acute kidney injury, chronic kidney disease, renal dysfunction, and perioperative renal outcomes. To improve completeness, the reference lists of key publications were manually screened for additional relevant articles. The final selection prioritized AWR-specific clinical studies, mechanistically informative reports, and perioperative literature with direct relevance to the interpretation of renal vulnerability in complex ventral hernia repair. Given the still limited number of AWR-specific renal studies, the purpose of this review was not to claim a definitive evidentiary model, but to provide a clinically useful synthesis of the data currently available and to identify the perioperative scenarios in which renal risk appears most relevant to the abdominal wall surgeon.

### Why complex abdominal wall reconstruction carries a distinctive renal risk

**Fig. 1:**
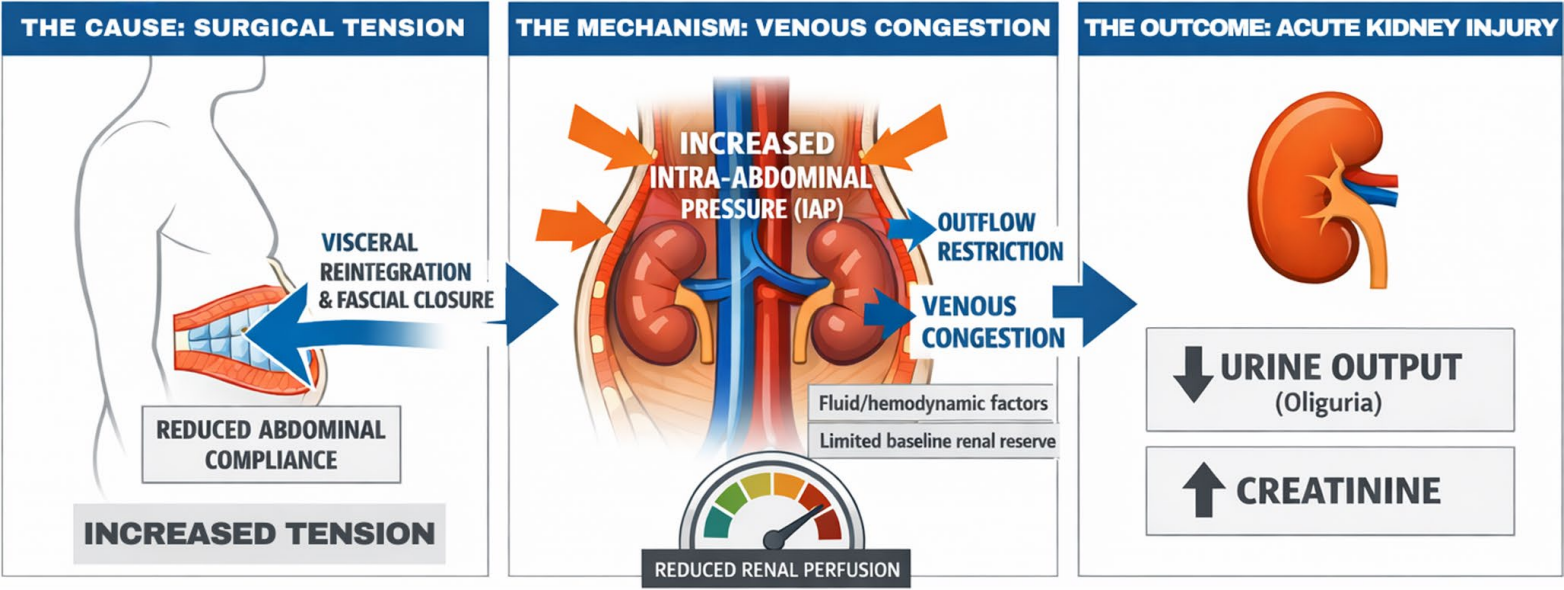
Proposed pathophysiological cascade linking complex abdominal wall reconstruction to postoperative acute kidney injury. Visceral reintegration and fascial closure may reduce abdominal compliance and increase intra-abdominal pressure, particularly in high-tension repairs [2, 6, 8, 10]. The resulting pressure-related stress may impair renal venous outflow, promote venous congestion, and reduce renal perfusion [10–13]. Perioperative fluid/hemodynamic factors and limited baseline renal reserve may amplify this process, culminating in oliguria, rising serum creatinine, and postoperative acute kidney injury [4, 5, 14, 15]. This figure is intended as a conceptual framework rather than a validated risk model

Complex AWR is not simply another laparotomy with fascial closure [2, 6, 8]. In patients with loss of domain, reconstruction reverses a chronic state of visceral exteriorization and reduced intra-abdominal content accommodation, forcing the abdominal cavity into a new pressure–volume relationship for which neither the abdominal wall nor the cardiopulmonary-renal axis is fully adapted [6, 9]. By reintegrating viscera and reapproximating the fascia—often with TAR—complex AWR creates a postoperative setting in which reduced compliance, increased intra-abdominal pressure (IAP), impaired venous return, and reduced renal perfusion may become directly linked [2, 6, 8, 10]. For the surgeon, this means that postoperative oliguria may be a mechanical and hemodynamic warning sign, not merely a volume signal [2, 4, 6]. The proposed pathophysiological cascade linking reconstructive tension, increased intra-abdominal pressure, venous congestion, and postoperative acute kidney injury is summarized in Fig. 1.

The central mechanism is the abrupt loss of abdominal compliance imposed by visceral reintegration and fascial closure [2, 6, 8]. Once this buffering capacity is reduced, even modest fluid shifts may translate more readily into postoperative IAP elevation [10, 16]. In complex repairs with TAR, this phenomenon has been documented intraoperatively, with median IAP rising from 5 to 9 mmHg after closure together with increased airway plateau pressures, providing objective evidence that reconstruction itself generates measurable compartmental stress [17, 18]. Clinically, the issue is not pressure alone, but what that pressure does to organ perfusion [10–13]. Higher IAP can increase renal venous pressure, reduce effective transrenal perfusion pressure, and promote a combined phenotype of venous congestion and reduced inflow, particularly in patients with limited reserve [11–13].

This insult may be amplified by reduced venous return and secondary reductions in cardiac output, so that the postoperative phenotype can combine congestion and ischemia even when overt hypotension is absent [10, 14, 19]. This is precisely why a “normotensive” patient after difficult AWR may still be physiologically underperfusing the kidney [10, 11, 14, 20]. Recent IAH literature further supports this interpretation by showing that even modest pressure elevations can impair venous return and organ perfusion, while unnecessary fluid expansion may aggravate the pressure-perfusion mismatch rather than correct it [19, 21, 22]. In that sense, AWR occupies a distinctive position within the spectrum of IAH and ACS: it is not a spontaneous compartment problem, but a surgically induced form of compliance impairment arising directly from the reconstructive act itself [6, 11].

The mechanistic relevance of this model is illustrated by the report of Hasan and Sorensen, in which complex AWR was followed by severe ACS with oliguria, hyperkalemia, elevated airway and bladder pressures, and acute renal failure that reversed after nonoperative reduction of abdominal pressure, without undoing the repair [23]. This case should not be read as representative of typical AWR, but as proof that the reconstructed abdominal wall can, under conditions of severe compliance impairment, become the direct driver of renal and respiratory dysfunction through a pressure-mediated pathway [6, 23, 24].

The available epidemiologic data position AWR as a setting of substantial renal vulnerability, even under elective conditions and in apparently optimized patients [4, 5, 25]. Postoperative AKI occurs in 6% to 21% of patients undergoing complex ventral hernia repair, with rates varying according to defect extent, operative technique, and baseline renal function [4, 5, 25]. Of particular relevance, TAR has been associated with higher odds of AKI compared with retrorectus repairs without muscular release; however, TAR should be interpreted less as an isolated causal label and more as a marker of reconstructive intensity, tension redistribution, and closure difficulty in anatomically extreme repairs [25, 26]. In patients with pre-existing CKD, AKI rates after elective AWR with TAR exceed 26%, reinforcing that the already compromised kidney lacks sufficient functional reserve to tolerate the hemodynamic perturbations imposed by reconstruction [5, 15]. Taken together, these data support the view that complex AWR represents a setting of nontrivial renal vulnerability, particularly when reconstructive intensity and limited baseline renal reserve coexist [4, 5, 25].

### How reconstruction can precipitate renal dysfunction

Renal dysfunction after complex AWR is best understood as the result of converging mechanical, hemodynamic, and host-related stresses [10, 14, 21, 27]. Visceral reintegration and fascial closure reduce abdominal compliance and favor postoperative IAP elevation, whereas operative duration, blood loss, third spacing, vasodilation, transfusion, and fluid strategy determine how much effective renal perfusion remains available within this altered compartmental environment [10, 14, 21]. Accordingly, oliguria after complex reconstruction should not be interpreted reflexively as isolated hypovolemia, because it may also reflect venous congestion, extrinsic renal compression, and pressure-mediated reduction in renal perfusion [11–13].

Host reserve determines how much of this insult becomes clinically manifest [15, 28, 29]. Patients with CKD, diabetes, hypertension, obesity, frailty, and older biological age are less likely to tolerate the combined effects of pressure stress, hemodynamic instability, and inflammation [15, 28–30]. In these patients, complex AWR may not create an entirely new renal problem so much as expose a limited capacity to buffer reconstructive stress [5, 15, 28, 29].

### What the available clinical data mean for the abdominal wall surgeon

The clinical literature remains limited, but it is sufficiently coherent to justify procedure-specific attention [4, 5, 25]. Although the current evidence base is not yet large enough to support definitive risk models, it is already adequate to support a surgeon-oriented interpretation of where renal vulnerability emerges in complex AWR and why it should not be dismissed as incidental [4, 5, 25]. Across the available cohorts, postoperative AKI appears enriched in the same patients surgeons already recognize as physiologically demanding: those undergoing large ventral hernia repair, TAR-level reconstruction, or surgery on a background of reduced renal reserve [4, 5, 25]. For a Hernia readership, the key message is therefore not merely that AKI occurs, but that it appears to track with the same anatomical and physiological complexity that already defines difficult AWR [4, 5, 25]. In other words, renal dysfunction is not separate from reconstructive difficulty; it may be one of its most clinically useful systemic readouts [4, 5, 25]. The selected AWR-specific studies that most directly inform this surgeon-oriented interpretation are summarized in Table 1.

**Table 1:** Selected AWR-specific studies informing the interpretation of renal vulnerability after complex abdominal wall reconstruction

Study	Study type and AWR context	Main renal/physiologic signal	Why it matters for this review
[2] Petro et al., 2015	Clinical AWR study evaluating postoperative pressure behavior after complex reconstruction.	Shows that measurable postoperative intra-abdominal pressure elevation may occur after complex AWR and requires context-specific interpretation.	Provides an AWR-specific pressure substrate that may contextualize postoperative oliguria and renal stress.
[17, 18] Espinosa-de-Los-Monteros et al., 2022; Oprea et al., 2021	Intraoperative physiologic studies in TAR-based complex ventral/incisional hernia repair.	After closure, intra-abdominal pressure and respiratory/airway indices increased, supporting the view that reconstruction itself can generate measurable compartmental stress.	Supports the biologic plausibility that closure mechanics contribute to pressure-mediated renal vulnerability.
[23] Hasan and Sorensen, 2013	Case report of complex AWR complicated by severe abdominal compartment syndrome.	Oliguria, hyperkalemia, elevated airway/bladder pressures, and acute renal failure reversed after abdominal pressure reduction without taking down the repair.	Extreme but persuasive clinical illustration of pressure-mediated renal dysfunction after AWR.
[4] Shelby et al., 2023	Cohort study of complex AWR with perioperative fluid-management analysis.	Postoperative AKI occurred in 21.2%; perioperative fluid management emerged as a potentially modifiable domain, without intervention-level proof of causality.	Shows that renal injury is clinically frequent enough to merit attention and suggests a link between risk and perioperative management.
[25] Schaeffer et al., 2022	Comparative cohort in large ventral hernia repair.	AKI risk was higher in TAR than in retrorectus repair without muscular release.	Associates renal risk with greater reconstructive intensity and anatomically extreme repair.
[5] Messer et al., 2026	Cohort study with postdischarge renal follow-up after large ventral hernia repair.	AKI 14.2%, new-onset CKD 6.9%, and CKD progression 19.6%; postoperative AKI independently predicted later renal deterioration. In patients with pre-existing CKD undergoing elective AWR with TAR, AKI exceeded 26%.	Provides the clearest available support for extending concern beyond the index admission and viewing AWR within the AKI-AKD-CKD continuum.

Abbreviations: *AWR* abdominal wall reconstruction, *TAR* transversus abdominis release, *IAP* intra-abdominal pressure, *AKD *acute kidney disease, *AKI* acute kidney injury, *CKD* chronic kidney disease

This table intentionally prioritizes selected AWR-specific studies that most directly inform renal interpretation. Not all included studies reported renal endpoints as primary outcomes, but all contribute clinically relevant physiologic or outcome data for surgeon-facing interpretation

Messer et al. further extended this discussion by showing that the renal relevance of AWR may not be confined to the index admission [5]. In their cohort, postoperative AKI occurred in 14.2%, new-onset CKD in 6.9%, and CKD progression in 19.6%, with postoperative AKI emerging as an independent predictor of subsequent renal deterioration [5]. Although current data do not prove that AWR directly causes CKD progression, they do support a pragmatic interpretation for surgeons: postoperative AKI after complex AWR may identify the subgroup that deserves closer renal follow-up beyond discharge, particularly when baseline CKD, TAR, or major visceral reintegration are present [5, 7, 31, 32]. In this context, renal dysfunction after AWR may be better understood within the AKI–AKD–CKD continuum proposed by ADQI [5, 7, 31]. This longitudinal perspective is particularly relevant in patients with pre-existing CKD, TAR-level repairs, or early postoperative AKI, in whom inpatient creatinine surveillance alone is unlikely to capture the full renal burden of reconstruction [5, 25, 31, 32].

From a practical surgical standpoint, the key renal messages in complex AWR can be summarized as follows:

### Box 1. What the abdominal wall surgeon should know about renal risk in complex AWR

After complex AWR, oliguria should not be assumed to represent hypovolemia alone [10, 21, 33]. Loss of domain, substantial visceral reintegration, and TAR increase the need for pressure-aware postoperative interpretation [9, 25, 26]. CKD, albuminuria, diabetes, obesity, and frailty reduce tolerance to reconstructive stress [15, 28, 29]. Abdominal distension, marked wall tension, hyperkalemia, worsening respiratory mechanics, rising airway pressures, or elevated bladder pressure should raise concern for pressure-mediated organ dysfunction [10, 23, 33, 34]. Postoperative AKI should not be dismissed as transient, because it may identify patients at risk for later renal decline [5, 7, 31, 32].

This is precisely why the current literature is already clinically useful even before it becomes methodologically definitive: it consistently identifies where the surgeon should be more alert, even if it does not yet provide a fully validated prognostic architecture [4, 5, 25]. This practical interpretive value also supports the pressure–volume–reserve framework proposed below [2, 33].

A practical framework for renal risk after complex AWR: pressure, volume, and reserve for the abdominal wall surgeon, renal risk after complex AWR may be interpreted through three interacting domains: pressure, volume, and reserve [2, 5, 33]. Pressure refers to reduced abdominal compliance, visceral reintegration in loss-of-domain settings, fascial closure under tension, and postoperative IAP rise, along a continuum that may range from transient oliguria to phenotypes approaching intra-abdominal hypertension (IAH) and abdominal compartment syndrome (ACS) [10, 33]. In this context, the World Society of the Abdominal Compartment Syndrome continues to define IAH as sustained or repeated IAP ≥ 12 mmHg, and ACS as pressure > 20 mmHg associated with new organ dysfunction [10]. Volume refers to blood loss, third spacing, vasodilation, transfusion, under-resuscitation, unnecessary fluid expansion, venous congestion, and hemodynamic instability, all of which influence how the reconstructed abdomen is physiologically tolerated and are recognized contributors to postoperative AKI in major non-cardiac surgery [4, 10, 14, 21, 22].

Reserve refers to the patient’s capacity to tolerate reconstructive stress before the first incision is made [15, 28, 29]; in practical terms, this includes CKD, albuminuria, diabetes, hypertension, obesity, frailty, and advanced age, all of which reduce tolerance to the pressure-hemodynamic burden imposed by major AWR [15, 28, 29]. This should be presented explicitly as a clinical reasoning tool rather than a score, intended to help the surgeon interpret difficult postoperative physiology in anatomically extreme repairs [2, 33].

Operationally, this framework can be translated into four overlapping clinical phenotypes: pressure-dominant dysfunction; volume/hemodynamic dysfunction; CKD-unmasking dysfunction; and AKI-to-CKD transition after discharge [5, 31, 35].

### What the surgeon should do before and after complex abdominal wall reconstruction

Preoperative renal risk assessment in AWR should be surgically contextualized [28, 36]. Reduced eGFR, CKD, and albuminuria remain important markers of limited reserve, but they should be interpreted together with variables that matter specifically in abdominal wall reconstruction—particularly loss of domain, the anticipated burden of visceral reintegration, and the need for TAR or other component separation techniques—because these features help determine how much postoperative pressure-related stress the repair is likely to generate [5, 9, 28]. Frailty, obesity, diabetes, and hypertension further identify the patient in whom an anatomically successful repair may still carry disproportionate physiologic cost [29, 36].

In selected patients with very large defects or marked loss of domain, preoperative compliance-modifying adjuncts may also be considered as part of the reconstructive plan [26, 37, 38]. At present, however, these tools should be framed as anatomy- and closure-oriented strategies rather than kidney-protective interventions per se, because contemporary evidence remains mixed and direct renal benefit has not been demonstrated [26, 37, 38].

The goal is not to create a separate renal pathway or a competing ERAS protocol, but to improve perioperative interpretation within standard complex AWR care [3, 4]. Fluid management should avoid both under-resuscitation and reflexive liberal expansion [3, 4, 21, 22]. In complex AWR, postoperative oliguria should not automatically trigger repeated fluid loading, because it may reflect pressure-mediated organ dysfunction as much as true hypovolemia [4, 21, 22]. Early postoperative surveillance should therefore integrate urine output and creatinine with direct bedside suspicion for abdominal mechanical stress and, when that concern is substantive, objective pressure assessment rather than repeated empiric fluid loading alone [10, 33]. This is particularly relevant when oliguria coexists with abdominal distension, marked wall tension, hyperkalemia, worsening respiratory mechanics, rising airway pressures, or elevated bladder pressure when measured [10, 23, 33, 34].

The patients most likely to benefit from intensified renal awareness are those in whom host vulnerability and reconstructive complexity coexist [5, 9, 25, 28, 29]. In practical terms, this means the patient with CKD, albuminuria, diabetes, frailty, or obesity who is also undergoing large ventral hernia repair with loss of domain, substantial visceral reintegration, or TAR-level reconstruction [5, 9, 25]. In these settings, renal risk should not be inferred from baseline creatinine alone, because the postoperative burden arises from the interaction between reduced reserve and the mechanical consequences of reconstruction [5, 9, 28]. This does not imply a separate renal pathway for every ventral hernia repair. Rather, it argues that in the anatomically and physiologically extreme AWR patient, the surgeon should interpret oliguria, fluid responsiveness, abdominal tension, and respiratory deterioration as potentially linked events rather than isolated postoperative findings [4, 6, 33].

### What the field still needs: better surgeon-relevant renal data in complex AWR

Current AWR studies have already identified meaningful renal signals, but most remain centered on AKI during the index admission and still fall short of an integrated model linking hernia anatomy, reconstructive strategy, postoperative pressure biology, perioperative management, and longer-term renal outcomes [2, 4, 5]. This should be interpreted not as evidence against the clinical relevance of renal risk in complex AWR, but as evidence that the field has advanced far enough to define the problem clearly, while not yet far enough to resolve it definitively [2, 4, 5].

Three priorities appear most relevant. First, renal events should be defined using standardized KDIGO-based AKI criteria and, when feasible, extended beyond the index admission to AKD and later CKD decline [7, 31, 39]. Second, hernia-specific variables such as loss of domain, visceral reintegration burden, and component separation should be analyzed as physiologic determinants rather than merely technical descriptors [2, 5, 9]. Third, prospective multicenter studies should test whether pressure-aware perioperative strategies can better identify patients at risk and improve postoperative management in complex AWR [3, 10, 33].

Prospective datasets should also capture bladder-pressure trajectories, compliance-modifying adjuncts, and postdischarge renal follow-up, so that anatomy, technique, pressure biology, and longitudinal renal outcomes can be examined within the same analytic framework [5, 34, 37]. Whether structural kidney injury biomarkers could help identify early pressure-mediated renal stress after complex AWR also remains unknown [40]. Although damage and stress biomarkers such as NGAL and TIMP-2·IGFBP7 have been increasingly studied in AKI more broadly, including perioperative settings, their role has not been specifically defined in the abdominal wall reconstruction literature [40].

## Conclusions

Complex AWR should be recognized not only as an anatomic operation, but also as a reconstruction with important physiologic consequences [2, 5, 8]. The available evidence, although still limited in volume, consistently suggests that postoperative renal dysfunction after complex reconstruction is not incidental: it appears to emerge from the interaction between visceral reintegration, reduced abdominal compliance, altered pressure biology, perioperative fluid/hemodynamic management, and baseline renal reserve [2, 4, 5].

For abdominal wall surgeons, the main implication is practical [4, 6]. In the patient with loss of domain, major visceral reintegration, TAR-level complexity, or limited preoperative renal reserve, postoperative oliguria should not be interpreted simplistically, and renal surveillance should be more deliberate [4, 6, 9, 28]. Recognizing the kidney as part of modern AWR physiology does not compete with reconstructive goals; it helps the surgeon recognize when an anatomically successful repair may be carrying disproportionate physiologic cost [4, 6].

## Data Availability

No datasets were generated or analysed during the current study.
